# Dandelion root extract affects colorectal cancer proliferation and survival through the activation of multiple death signalling pathways

**DOI:** 10.18632/oncotarget.11485

**Published:** 2016-08-22

**Authors:** Pamela Ovadje, Saleem Ammar, Jose-Antonio Guerrero, John Thor Arnason, Siyaram Pandey

**Affiliations:** ^1^ Department of Chemistry & Biochemistry, University of Windsor, Windsor ON, Canada; ^2^ Department of Biology, University of Ottawa, Ottawa ON, Canada; ^3^ Red de Estudios Moleculares Avanzados, Instituto de Ecología A.C. Xalapa, Veracruz, México

**Keywords:** cancer, oxidative stress, phytochemical composition, gene expression, natural health product

## Abstract

Dandelion extracts have been studied extensively in recent years for its anti-depressant and anti-inflammatory activity. Recent work from our lab, with *in-vitro* systems, shows the anti-cancer potential of an aqueous dandelion root extract (DRE) in several cancer cell models, with no toxicity to non-cancer cells. In this study, we examined the cancer cell-killing effectiveness of an aqueous DRE in colon cancer cell models. Aqueous DRE induced programmed cell death (PCD) selectively in > 95% of colon cancer cells, irrespective of their p53 status, by 48 hours of treatment. The anti-cancer efficacy of this extract was confirmed in *in-vivo* studies, as the oral administration of DRE retarded the growth of human colon xenograft models by more than 90%. We found the activation of multiple death pathways in cancer cells by DRE treatment, as revealed by gene expression analyses showing the expression of genes implicated in programmed cell death. Phytochemical analyses of the extract showed complex multi-component composition of the DRE, including some known bioactive phytochemicals such as α-amyrin, β-amyrin, lupeol and taraxasterol. This suggested that this natural extract could engage and effectively target multiple vulnerabilities of cancer cells. Therefore, DRE could be a non-toxic and effective anti-cancer alternative, instrumental for reducing the occurrence of cancer cells drug-resistance.

## INTRODUCTION

The increases in the world's aging population, as well as the adoption of cancer-causing behaviors, are the major contributors to a global escalation of different forms of cancers. There are over 12 million new cancer cases arising annually and over 7 million cancer-related deaths worldwide, and even with the introduction of many chemotherapy and chemopreventive approaches, cancer is still one of the leading causes of deaths in the world today, with a statistic of one in four deaths being attributed to cancer alone [1, 2].

Despite the progress made in the development and introduction of many chemotherapy agents, the accompanying toxicities and side effects [1], indicate that further research is required to reduce the incidence of cancer rates, and the amount of cancer-related deaths, as well as to improve the quality of life of patients already diagnosed with the disease.

Natural health products (NHPs) and natural products (NPs) have been essential in the development of many drugs, with over 75% of the currently available chemotherapies having been derived from natural sources (plants, microbes and marine sources), with a common example being paclitaxel [3]. These NHPs have been used in various traditional medicines and recent studies on the use of the NHPs for specific diseases yield some scientific validation for their application [4, 5]. Even with all the incoming evidence, herbal drugs and other NHPs and NPs are usually shunned during systemic chemotherapy because of a possible herb-drug interaction that might enhance chemotherapy-related toxicity [6, 7].

Dandelions (*Taraxacum spp*) have been used for centuries for the treatment of various ailments; surprisingly enough, they have received little research attention. Some scientific studies report anti-inflammatory, anti-oxidative and diuretic activities of various parts of this plant [8]. Recent studies from our lab show a strong anti-cancer activity of an aqueous dandelion root extract (DRE) [9, 10, 11, 12]. We find that DRE is able to induce a rapid activation of the death-receptor mediated extrinsic pathway of apoptosis in human leukemia and pancreatic cancer cells in a dose and time dependent manner. Furthermore, the induction of apoptosis is dependent on caspase-8 activation. Our data shows that this action of DRE is cancer cell selective, as the same treatment is not detrimental to non-cancer cells. However, the detailed analyses of the efficacy and toxicity of this extract in *in-vivo* and *ex-vivo* models, as well as, its mechanism(s) of action still remain unexplored. Furthermore, the pharmacologically active anti-cancer components of this extract are at present unknown.

We report the anti-cancer activity of the DRE obtained with *in-vitro* (colon cancer cell lines) and *in-vivo* (mouse xenograft model of colon cancer) models. We hypothesized that due to its compositional complexity (mixture of bioactives), DRE might be able to activate different signaling events and more efficiently induce program cell death (PCD) processes by targeting different metabolic vulnerabilities of cancer cells. Accordingly, we have shown that, although DRE treatment triggered cell death in all cell models examined and led to the activation and localization of active caspase-8 to the mitochondria and the peri-nuclear space, this caspase-8 activation was not essential for the induction of cell death in colon cancer cells as an inhibition of caspase-8 activation did not alter the cytotoxicity of DRE. Therefore, in colorectal cancer cells the DRE treatment must have utilized caspase-8 independent cell death pathway. We have been able to identify four pharmacologically active components, α-amyrin, β-amyrin, lupeol and taraxasterol, in two out of the six bioactive fractions, but the anti-cancer activities of the individual compounds were not as strong as that of the unfractionated DRE indicating, clearly, the benefits of using the whole extract. Taken together our results scientifically validate the use of NHPs, especially dandelion root extracts, as potential anti-cancer agents, which might represent a novel non-toxic alternative to conventional cancer therapy available today.

## RESULTS

### Dandelion root extract (DRE) induces apoptosis in aggressive colorectal cancer cells

**Figure 1:**
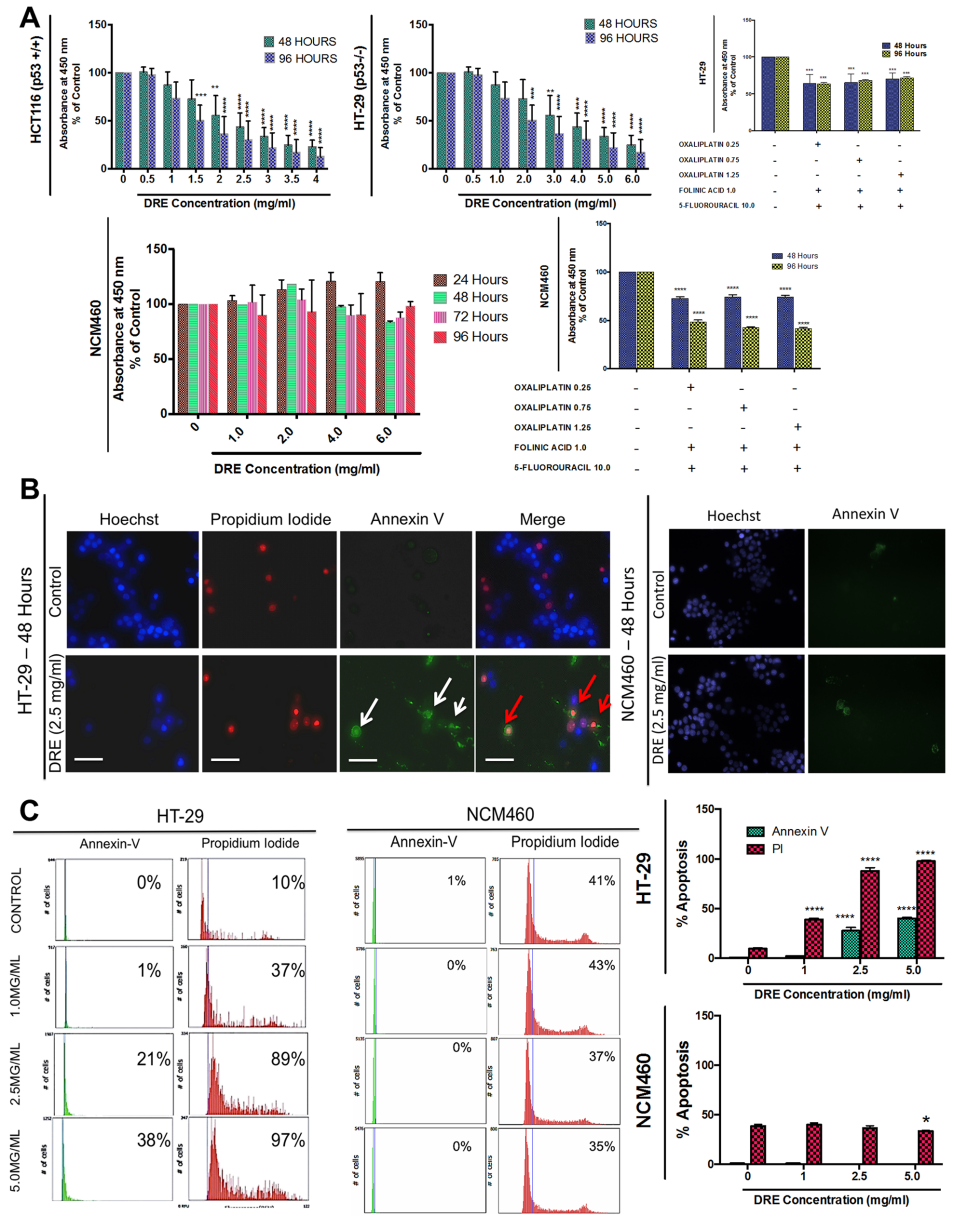
Dandelion root extract induces apoptosis in aggressive colorectal cancer cells Colon Cancer cells (HT-29 [p53−/−] and HCT116 [p53+/+]) and normal colon mucosal epithelial cells (NCM460) were treated with increasing doses of DRE and analyzed for anticancer effects. **A.** Viability of colon cells treated with DRE. Results are expressed as mean ± SD from quadruplicates of 3 independent experiments. **P<0.001, ****P<0.0001 **B.** Hoechst (Blue), Annexin V (Green) and Propidium Iodide (PI) (Red) staining of DRE-treated cells, 48 hours after treatment to assess induction of apoptosis. Fluorescence images were obtained at 400X magnification; Scale bar = 25 μm. Apoptotic cells were quantified using image-based cytometry **C.** *P<0.05, ****P<0.0001.

This apoptosis-inducing activity of DRE, as previously reported [9, 11] prompted further studies into its efficacy in highly aggressive colorectal cancer cells, HT-29 (p53−/−) and HCT116 (p53 WT). For comparison, normal colon mucosal epithelial cells (NCM460) were also used to assess the selectivity of DRE to colorectal cancer cells. Furthermore, we compared the efficacy of DRE to the currently utilized colon cancer chemotherapy, FOLFOX (5-fluorouracil, Folinic Acid and Oxaliplatin). The results are summarized in Figure 1. We observed a significant decrease in the viability of both HT-29 and HCT116 colorectal cancer cells following the DRE treatment. This effect was both time and dose dependent and it was similar in both cell lines, irrespective of their p53 status. Employing the WST-1 cell viability assay, we determined the EC_50_ of DRE in both colon cancer cell lines; 2.0 mg/ml in HCT116 cells and 3.5 mg/ml in HT-29 cells. The selectivity of DRE to cancer cells was once again confirmed, as normal NCM460 cells were DRE refractive and did not lose metabolic activity and cell viability when exposed to the same doses and time points as the colon cancer cells. Furthermore, the efficacy and selectivity of DRE to colorectal cancer cells was compared to that of FOLFOX. It was observed that the FOLFOX combination did not have a selective effect to colorectal cancer cells, as the normal colon mucosal epithelial cells were also affected at the same doses (Figure 1A).

This reduction in metabolic viability corresponded to an increase in apoptosis induction, as DRE treatment triggered apoptosis selectively in colon cancer cells, but not in normal mucosal cells, which was subsequently confirmed by fluorescence microscopy following Hoechst 33342, propidium iodide and Annexin V staining, to observe the nuclear morphology, cell membrane integrity and externalization of phosphatidylserine respectively. In the DRE-treated colon cancer cells we observed significant increases in propidium iodide and Annexin V positive staining, indicative of apoptosis, while NCM460 cells, again, remained unaffected (Figure 1B). Image-based cytometry was used to quantify the apoptotic response and the data showed an approximately 40% increase in Annexin V positive cells and a corresponding 97% increase in propidium iodide staining in DRE responsive cells (Figure 1C). These results confirmed the anti-cancer potential of DRE and demonstrated its efficacy in aggressive colorectal cancer cells regardless of their p53 status.

### Dandelion root extract selectively impairs the migration of colon cancer cells

**Figure 2:**
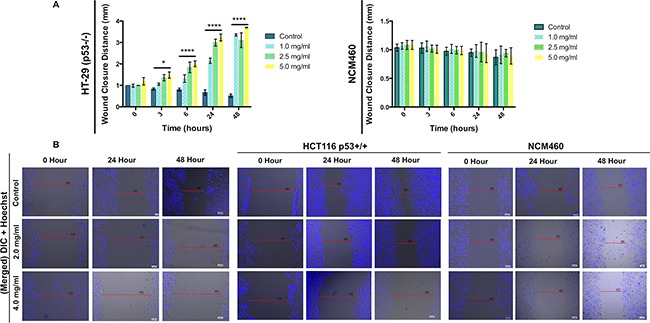
Dandelion root extract impairs the migration of colorectal cancer cells **A.** Quantification of wound closure distance in HT-29 and NCM460 cells at different treatment times (0, 3, 6, 24 and 48 hours). Values are expressed as mean ± SD **P<0.001, ****P<0.0001. **B.** Representative micrographs of wound healing assay in HT-29, HCT116 and NCM460 cells.

To determine if DRE can prevent invasive and metastatic behaviours in colorectal cancer cells, the scratch wound healing assay was employed. HT-29, HCT116 and NCM460 cells were pre-treated with thymidine for 18 hours to halt further proliferation, following which, the cells were treated with DRE at the indicated concentrations. Cells were monitored at 0 hours (at the time of treatment) and at 3, 6, 24 and 48 hours following treatment (Figure 2). It was observed that treatment with DRE inhibited the ability of colorectal cancer cells, HT-29 and HCT116, to migrate into the wound, unlike the control untreated samples that freely migrated into the wound area. As anticipated, the normal NCM460 cells treated with DRE were able to migrate into the scratch wound area (Figure 2), confirming the selectivity of DRE to cancer cells. These results clearly indicate that dandelion root extract can inhibit the ability of colorectal cancer cells to migrate and invade, and therefore metastasize to secondary locations.

### Dandelion root extract retards the growth of human colon tumors in mouse xenograft models

As described above, the *in-vitro* studies proved that DRE induced selective apoptosis in cancer cells; thus distinguishing between cancerous and non-cancerous cells. The next question was whether DRE was safe to use *in-vivo* and whether it could be effective against tumors grown as xenografts.

**Figure 3:**
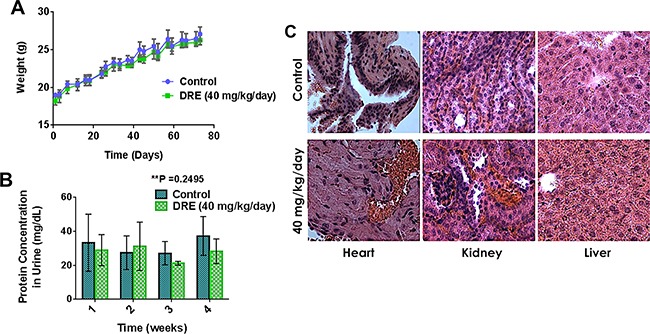
Dandelion root extract is well tolerated in animal models Balb/C mice were separated into two groups, one group on oral administration of PBS – control and the other group on oral administration of DRE (40 mg/kg/day) for a period of 75 days. **A.** Weight of mice in each group for the duration of the study. **B.** Urine was obtained from mice every week for the last 4 weeks of the study. Protein urinalysis was carried out using urine dipstick and the Bradford protein assay. **C.** Hematoxylin and Eosin staining of tissues (hearts, kidneys and livers). Images were obtained on a brightfield microscope at 63X Objective.

The first step in this part of the study was to assess the safety of systemic DRE administration to normal Balb/c mice, at a dose of 40 mg/kg/day, for a period of 75 days. The mice were observed for any signs of toxicity during this time period, by weight measurement and protein urinalysis. There were no observable differences between the control-untreated and the DRE-treated groups in weight gain (Figure 3A) or protein content in urine (Figure 3B), indicating a lack of toxicity. The protein urinalysis identified trace amounts of protein in the urine of mice from both groups, without any significant differences between the groups (Figure 3B).

After the completion of 75 days safety study, histopathological analysis, by Dr. A. Brooke was performed on tissues obtained from the mice. Hematoxylin and eosin staining of the hearts, livers and kidneys showed no gross morphological differences in tissue slices between the control untreated and the DRE treated group (Figure 3C). The tissue lesions were minimal or mild and were interpreted as either background or incidental and none was of a type or frequency indicative of DRE toxicity to the Balb/c mice (Table 1). Taken together, these results established that systemic oral intake of the DRE was safe and its anti-cancer efficacy should be further investigated. This was done using mouse xenografts of colon cancer.

**Table 1:** Summary of histological lesions in normal balb/c mice on DRE regimen

	No Treatment (Plain-filtered water)					Treatment group (DRE-Supplemented water)			
	M1	M2	M3	M4	M5	M1	M2	M3	M4
**Liver:**									
-Infiltration, leukocyte, predominantly mononuclear, minimal		**X**	**X**		**X**				
-Focal mineralization, minimal						**X**			
-Hepatocyte necrosis, minimal									
-Focus of cellular alteration, eosinophilic, minimal			**X**	**X**		**X**			
-Hepatocyte vacuolation, lipid type, minimal			**X**	**X**				**X**	
-Hepatocyte vacuolation, lipid type, mild	**X**			**X**		**X**			**X**
Fibrin thrombus			**X**						
**Heart:**									
-Infiltration, leukocyte, predominantly mononuclear, minimal		**X**				**X**			
Myofiber separation and vaculation, minimal (suspect artifact)		**X**	**X**					**X**	
**Kidney:**									
- Infiltration, leukocyte, predominantly mononuclear, minimal	**X**	**X**		**X**				**X**	**X**
Tubule vacuolation, minimal					**X**		**X**		**X**
Fibrin or other extracellular matrix, glomerulus									

The type, frequency and grade of histological lesions are summarized in this table. The histological lesions are minimal or mild and are interpreted as either background lesions or incidental lesions. X-indicates the mice containing different lesions.

**Figure 4:**
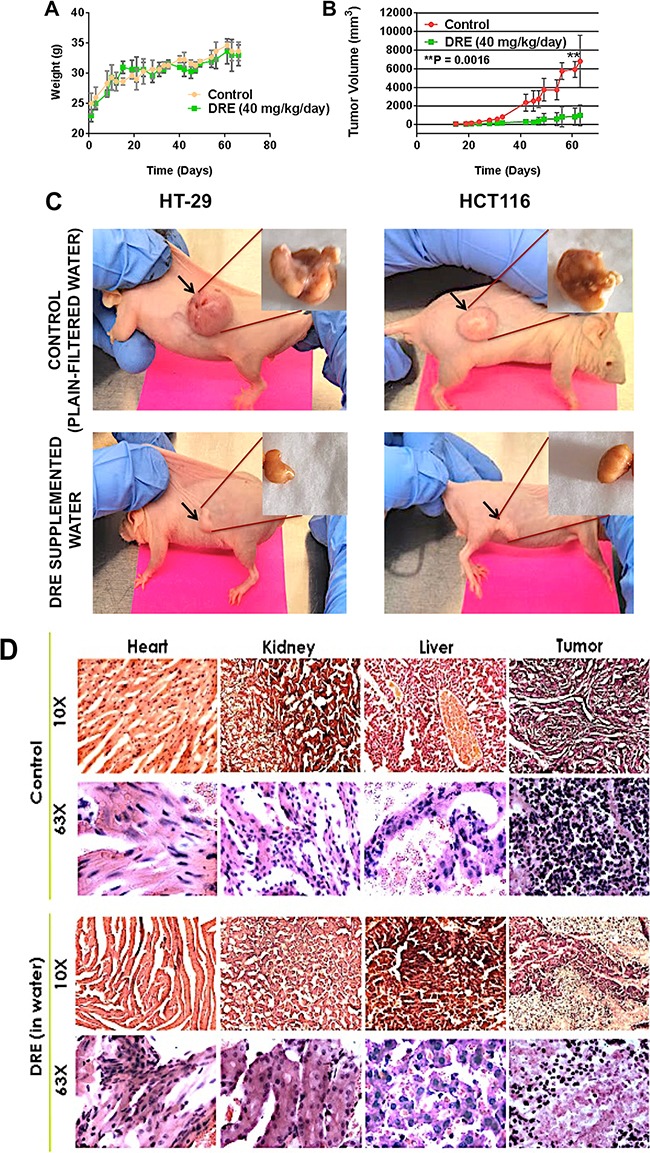
Dandelion root extract halts the growth of colon tumor xenografts Immunocompromised CD-1 nu/nu mice were subcutaneously injected with 2 × 10^6^ colon cancer cells (HT-29 on the left flank and HCT116 on the right flank). **A.** Mice weights every other day. **B.** The tumor volumes were measured using a standard caliper and the tumor volumes were calculated according to the formula π/6 × length × width. **C.** Images of mice tumors week 9 of the study, showing differences in the tumor sizes between the control, untreated group and the DRE group. **D.** Hematoxylin and Eosin staining of tissues. Images were obtained on a brightfield microscope at 63X Objective.

CD-1 nu/nu immunocompromised mice were subcutaneously injected with HT-29 p53−/− cells (left flank) and HCT116 p53 WT cells (right flank). Following the establishment of colon tumors, mice were separated into two groups – an untreated control group and a DRE treatment group (same groups as in the toxicity studies). Mice were observed for the duration of 75 days, while the weights and tumor volumes were measured three times a week. The obtained results demonstrated that oral administration of 40 mg/kg/day of DRE did not have any effects on the weights of the mice (Figure 4A) (as observed in the toxicity studies), but the DRE treatment efficiently suppressed the growth of both p53 WT and p53 mutant tumors *in-vivo* (Figure 4B – 4C). Additionally, H & E staining of the tumor revealed fewer nuclei in the DRE treated group, compared to the control group, and as expected, there were no gross morphological differences in the livers, kidneys and hearts of the control and DRE treated groups (Figure 4D). These results clearly indicated that the oral administration of DRE was not toxic in mice and was efficacious in halting the growth of colon tumors in xenograft models.

### Dandelion root extract targets the mitochondria of colon cancer cells

**Figure 5:**
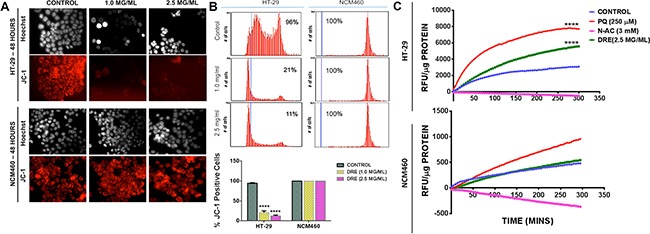
Dandelion root extract targets the mitochondria of colon cancer cells JC-1 staining on HT-29 and NCM460. Red fluorescence intensity was measured by fluorescence microscopy (Magnification: 400X) **A.** and image based cytometry **B.** to detect the loss of the mitochondrial membrane potential. **C.** ROS production was measured in isolated mitochondria. Fluorescence readings were taken in 5-minute intervals for 5 hours at Ex. 560 nm and Em. 590 nm and expressed as relative fluorescence units (RFU) per μg protein. Statistics – Two way ANOVA ****P<0.0001.

Few studies have attempted to delineate the mechanism of action of dandelion extracts, especially in cancer cells. Previously, we have shown that DRE does not induce DNA damage in cancer cells. However, as in the previous studies, we observed a disruption of the mitochondrial membrane potential following DRE treatment of colon cancer cells. On the other hand, the mitochondria of NCM460 cells remained unaffected (Figure 5). These responses were quantified and confirmed by image-based cytometry, which clearly showed a decrease in the intensity of red fluorescence, indicative of a loss of mitochondrial membrane potential, in HT-29 cells, with no difference between the control and DRE treated samples of NCM460 (Figure 5B). To further investigate the role of the mitochondria in DRE induced apoptosis, the mitochondria were isolated from HT-29 and NCM460 cells and treated directly with 2.5 mg/ml DRE, 250 μM Paraquat (PQ) or 3 mM N-acetylcysteine (N-Ac). In the presence of Amplex Red and HRP, the production of reactive oxygen species (ROS), such as H_2_0_2_ and superoxide can be measured. Fluorescence readings were taken every 5 minutes for a total duration of 4 hours. The obtained results showed a significant increase in the levels of ROS produced in the DRE-treated mitochondria of HT-29 cells, compared to the untreated controls and the antioxidant controls. It was also observed that the DRE treatment of isolated mitochondria from NCM460 cells did not produce any significant amounts of ROS (Figure 5C). Thus, it appears that DRE may target metabolic defect(s) and/or mitochondrial ROS response in cancer cells, to bring about selectivity for cancer cell killing.

### Activation of caspase-8 is not essential for cell death in human colon cancer cells

**Figure 6:**
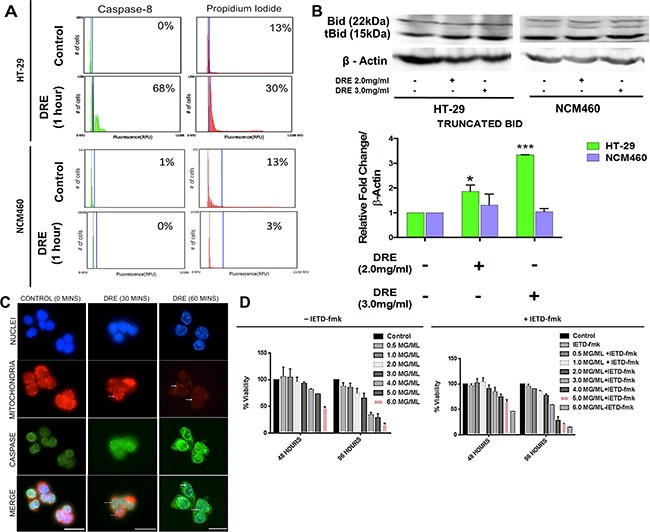
Dandelion root extract triggers the activation of caspase-8 **A.** Image-based cytometry of HT-29 and NCM460 cells stained with caspase-8 and incubated for an hour and counterstained with propidium iodide. **B.** Western blots and densitometry analysis of protein levels of truncated Bid. *P<0.05 **C.** Immunocytochemistry of activated caspase-8 and mitochondrial localization – MitoTracker (Red) and anti-caspase-8 antibody (Green), Hoechst 33342 (blue). Images were obtained at 400X objective. White arrows indicate the dispersion of MitoTracker Red, indicative of a loss of mitochondrial membrane potential and the magenta arrows show the presence of active caspase-8 in the nucleus following DRE treatment. Scale bars = 25 μm. **D.** Prior to DRE treatment, HT-29 cells were pre-treated with a caspase-8 specific inhibitor, IETD-fmk, for an hour at indicated concentrations and analyzed for cell viability.

Our previous studies showed that DRE treatment rapidly and efficiently activates the extrinsic pathway of apoptosis in leukemia, melanoma and pancreatic cancer cells and this process is dependent on the activation of caspase-8 as the cells with a dominant-negative Fas-Associated Death Domain (Dn-FADD) are unresponsive to the DRE treatment [12]. Here we examined whether similar activation of the extrinsic apoptotic pathway takes place in DRE-treated colorectal cancer cells. Using substrates specific fluorescent-labeled inhibitors of caspases (FLICA; DEVD – Caspase-3; IETD – Caspase-8), the activation of these caspases was monitored in HT-29 and NCM460 cells following the DRE treatment between 15 minutes to 48 hours. The results obtained by image-based cytometry showed a 68% increase in caspase-8 activity with a corresponding 30% increase in propidium iodide staining in HT-29 cells treated with 2.5 mg/ml DRE for an hour. Furthermore, this response was specific to HT-29 cancer cells, as NCM460 normal cells remained unaffected by the same treatment. The NCM460 cells did not activate caspase-8 and did not uptake propidium iodide (Figure 6A). As mitochondrial membrane destabilization was observed in colon cancer cells treated with DRE (Figure 5A – 5B), we wanted to confirm the link between caspase-8 activation and the loss of mitochondrial membrane potential. Following treatment, the cells were lysed and probed for the truncated pro-apoptotic protein, Bid. Bid is known to translocate to the mitochondria following cleavage and activation by activated caspase-8. This translocation to the mitochondria has been shown to play a role in the destabilization of the mitochondrial membrane [13]. DRE treatment led to the truncation of Bid in HT-29 cells selectively, with no increase in Bid truncation in NCM460 cells (Figure 6B).

Caspases are crucial for the initiation, propagation and execution of apoptosis [14], and therefore their activation and localization has been a focus of much research, with many conflicting reports. Some reports suggest that before their activation pro-caspases are located in the cytosol, and remain in the cytosol even after exposure to the apoptotic stimuli and activation [14]. Others suggest that following activation, caspases re-localize to the mitochondria, where they interact with other pro-apoptotic proteins during the progression of apoptosis [15]. A third option, put forward by Qin and colleagues, suggests that inactive caspases are kept in the mitochondria, but following apoptotic stimuli and activation, they are released from the mitochondria into the cytoplasmic peri-nuclear space [16]. Here, we asked which of these three mechanistic scenarios the activation of Caspase-8 by the DRE treatment conformed to.

In order to do this, cells that were plated onto coverslips, treated with DRE for 30, 60, 180 and 360 minutes and the activation and localization of caspase-8 was examined. The cells were incubated with MitoTracker dye for 45 minutes, prior to immunocytochemical analysis as described in the materials and methods. The results showed a progressive destabilization of the mitochondrial membrane following the DRE treatment, which was observed as early as 30 minutes post treatment (Figure 6C). Pro-caspase-8 (green) was localized in the mitochondria (red) in control untreated cells; however, following the DRE treatment, activated caspase-8 was released from the mitochondria into cytoplasmic space, as indicated by the dispersed green fluorescence (Figure 6C). Therefore, DRE treatment efficiently activated the extrinsic pathway of apoptosis in colon cancer cells and the results confirm the localization of caspase-8 before and after activation. The question now was whether this activation of caspase-8 was the mandatory step in this process.

To establish this, cells were pre-incubated with a caspase-8 specific inhibitor, IETD-fmk for an hour, before the DRE treatment and the cells were analyzed by WST-1 viability assay. The results showed that cytotoxicity occurred even in the presence of the caspase-8 inhibitor, IETD-fmk, and the corresponding loss of HT-29 cells' viability in response to the DRE treatment was dose and time dependent (Figure 6D), suggesting that in HT-29 colorectal cancer cells the DRE-induced cell death was caspase-8 independent.

### Dandelion root extract promotes the expression of programmed cell death genes

**Figure 7:**
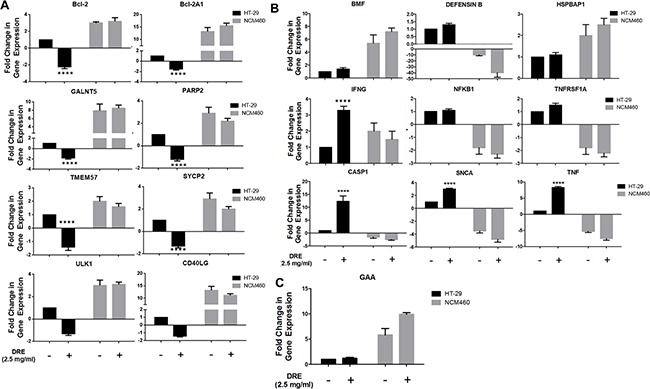
Differential fold change in expression of cell death genes in human colorectal cancer (HT-29) versus normal colon mucosal epithelial cells (NCM460) Differential expression levels of cell death genes affected by DRE treatment **A.** Anti-apoptotic genes **B.** Pro-apoptotic genes **C.** Pro-autophagic genes. The results are average of three independent experiments. *P<0.05 **** P<0.0001.

Since the DRE triggered cell death of colon cancer cells appeared to be caspase-8 independent, we proceeded to examine its effects on the expression cell death related genes in order to uncover other aspects of the DRE action. Using the RT^2^ Profiler Cell Death Pathway Finder PCR system, we analyzed the expression profiles of 84 key genes in the central mechanisms for apoptotic, autophagic and necrotic cell death to further determine the differences in colorectal cell response to DRE and to further investigate the selectivity of DRE to cancer cells, the gene expression study was carried out in HT-29 and NCM460 cells. Table 2 outlines the results obtained from this part of the study, emphasizing the differential expressions of genes between HT-29 colon cancer cells and NCM460 normal colon epithelial cells. It was observed that treatment with DRE had opposite effects in cancer and non-cancerous cells, when it came to how they express cell death genes, as cell death genes overexpressed in HT-29 cells following treatment, were down-regulated in NCM460 cells under the same conditions and vice versa (Figure 7). In this study, we found that DRE treatment led to the up-regulation of pro-death genes in HT-29 cells, including caspase 1, KCNIP1, SNCA, TNF and some of its corresponding receptors, TNFRSF1A and TNFRSF11B (Figure 7B. In parallel, we observe a down-regulation of pro-survival genes, especially anti-apoptotic genes, Bcl-2, Bcl-2A1 and PARP2. At the same time, the expression of these genes in non-cancerous NCM460 cells did not show a drastic change from the NCM460 control untreated cells (Figure 7A). Furthermore, where there were changes in the gene expression of NCM460 cells, the pattern was directly opposite to that of cancerous HT-29 cells. This increase in gene expression was not confined to one major pathway, as we observed increases in expressions of genes involved in apoptosis and autophagy, indicating that DRE hit multiple targets in cancer cells. This further confirmed the connections between the different forms of programmed cell death.

**Table 2:** Differential fold change in expression of cell death genes in human colorectal cancer (HT-29) versus normal colon mucosal epithelia cells

Anti-Apoptotic Genes			
Gene	Fold Change (HT-29)	Fold Change (NCM460)	Gene Function
Bcl-2 (B-Cell Lymphoma 2)	−2.2 ± 0.23	+3.2 ± 0.44	An integral outer mitochondrial membrane protein that blocks the apoptotic death by interacting and inhibiting pro-apoptotic proteins, e.g BAX, APAF1 [20]
Bcl-2A1 (Bcl-2 related protein A1)	−1.5 ± 0.21	+15.5 ± 1.1	BCL2A1 is a target of NF-kB activation, in response to inflammatory signals. This gene exerts pro-survival functions and is generally overexpressed in several cancers [21]
CD40LG	−1.4 ± 0.11	+11.2 ± 0.7	An integral membrane protein and a member of the TNF superfamily, overexpressed in several carcinomas. Enhances cytokine production and promotion of cell proliferation [22]
GALNT5 (polypeptide N-acetylgalactosaminyltransferase 5)	−1.8 ± 0.23	+8.5 ± 0.75	A member of the TNF superfamily. They are responsible for the altered O-linked glycosylation occurring during the development of various cancers and their progression via altering O-glycan biosynthesis [23]
PARP2 (poly (ADP-ribose) polymerase 2	−1.2 ± 0.15	+2.2 ± 0.25	Induced by double stranded DNA breaks, as a cellular response to DNA damage. It is involved in DNA repair and transcriptional regulation. PARP inhibitors are an emerging field in cancer therapy [24, 25]
SYCP2 (Synaptonemal Complex 2)	−1.3 ± 0.16	+2.0 ± 0.22	A major component of synaptic complexes during meiosis (prophase). May be involved in the organization of chromatin by temporarily binding to DNA scaffold attachment regions [26]
TMEM57 (Transmembrane Protein 57)	−1.4 ± 0.25	+1.6 ± 0.25	A target of Jun Kinase signaling. Has been shown to interact with several proteins, including the transcription regulators HTT and SMAD9). Its function is still being studied
ULK1 (Unc-51 Like Autophagy Activating Kinase 1)/ ATG1	−1.3 ± 0.16	+3.1 ± 0.2	A serine/threonine protein kinase involved in autophagy in response to starvation by phosphorylating Beclin-1. Transcriptional activation of ULK1 is involved in cancer cell survival [27, 28]
**Pro-Apoptotic Genes**			
BMF (Bcl-2 modifying factor)	+1.4 ± 0.23	+7.2 ± 0.55	A Bcl-2 family membrane that might activate apoptosis and anoikis. Interacts with other members of the Bcl-2 family for apoptosis induction [29, 30]
DEFB1 (Defensin, Beta 1)	+1.3 ± 0.09	−40 ± 7.54	Found to be down-regulated in several cancer types and plays a significant role in innate and adaptive immune response to promote cytotoxicity [31]
HSPBAP1 (Heat shock protein 27kDa associated protein 1)	+1.1 ± 0.11	+2.5 ± 0.3	May play a role in cellular stress response. Knockdown of this gene is associated with an increase in caspase-3/7 activation [32]
IFNG (Interferon Gamma)	+3.3 ± 0.25	+1.5 ± 0.5	Coordinate various cell response programs, including macrophage activation, inhibition of cell proliferation and activation of apoptosis [33]
NFKB1 (Nuclear factor kappa B)	+1.1 ± 0.11	−2.3 ± 0.3	Has both pro- and anti-apoptotic activities; a major transcription factor in various cell signaling pathways [34, 35]
TNFRSF1A (Tumor Necrosis Factor Receptor Superfamily, Member 1A)	+1.5 ± 0.15	−2.2 ± 0.3	Member of the TNF superfamily. Recruits adapter domains such as FADD and TRADD for activation of extrinsic apoptosis [36]
CASP1 (Caspase-1)	+12.3 ± 2.1	−2.5 ± 0.25	Cleaves a variety of substrates (121 substrates), including pro-inflammatory cytokine pro-interleukin (IL)-1β to induce apoptosis (pyroptosis) [37]
SNCA (Alpha synuclein)	+3.0 ± 0.14	−4.8 ± 0.45	A biomarker of colorectal cancer, and overexpressed in various other cancers; might be involved in cell death activation in certain cell types [38, 39]
TNF	+8.3 ± 0.32	−7.5 ± 0.55	A cytokine that binds to its receptors (TNFR1, TNFRSF1A) and promotes extrinsic apoptosis under ideal conditions [40]
**Pro-Autophagic Genes**			
GAA (Acid alpha glucosidase)	+1.2 ± 0.22	+9.9 ± 0.34	Active in the lysosomes for the breakdown of glycogen into glucose. Plays a role in autophaic induction by encouraging the degradation of p62 conjugated cargo [41]

**Figure 8:**
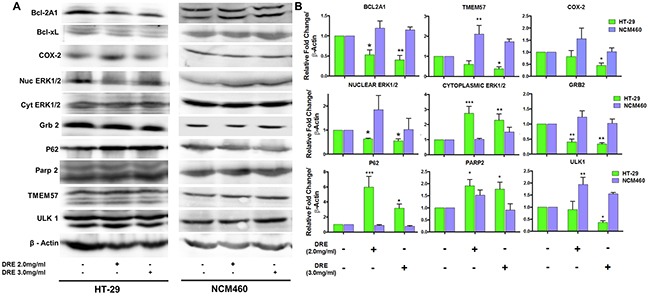
Activation of multiple signaling pathways by dandelion root extract **A.** Western blots of proteins involved in programmed cell death and cell survival and inflammation **B.** Densitometry quantification of western blot analysis from three independent experiments. *P<0.05, **P< 0.001.

The next step in further assessing the differential targeting of colorectal cancer cells was to determine if the changes in mRNA levels corresponded to changes in the protein levels. Not surprisingly, the protein levels, including Bcl-2A1, Bcl-xL, Parp-2, TMEM57 and ULK1 corresponded to the mRNA levels obtained during the gene expression analysis (Figure 8A). Furthermore, we wanted to assess other major players in colon cancer cell survival, including ERK1/2, Grb 2 and COX-2. We observed a decrease in nuclear ERK1/2 and a corresponding increase in the levels of cytoplasmic ERK1/2. This corresponds with findings that show that nuclear localization of ERK1/2 is essential for cell proliferation, while the cytoplasmic localization of ERK1/2 is required for the induction of apoptosis. Grb 2, an adaptor protein required for the activation of ERK1/2 that later translocates to the nucleus, was found to be decreased as well [17, 18] (Figure 8A – 8B). Cyclooxygenase-2 (COX-2) is a major promoter of colorectal cancer and is overexpressed in over 50% of colon cancers. This protein also has a major impact on various signaling pathways [19]. DRE treatment selectively decreased the expression of COX-2 in colon cancer cells in a dose and time dependent manner, showing its potential as an anti-inflammatory extract. These results showcase the versatility of dandelion root extract, which is able to target multiple programmed cell death pathways, as well as aberrant signaling pathways, selectively in colon cancer cells. This further shows that DRE was able to distinguish between cancer and non-cancer cells (colon) in order to achieve selective cytotoxicity to cancer cells, without associated toxicity to the non-cancer cells.

### Fractionation and phytochemical analysis of DRE

#### Identification of bioactive fractions & their components

**Figure 9:**
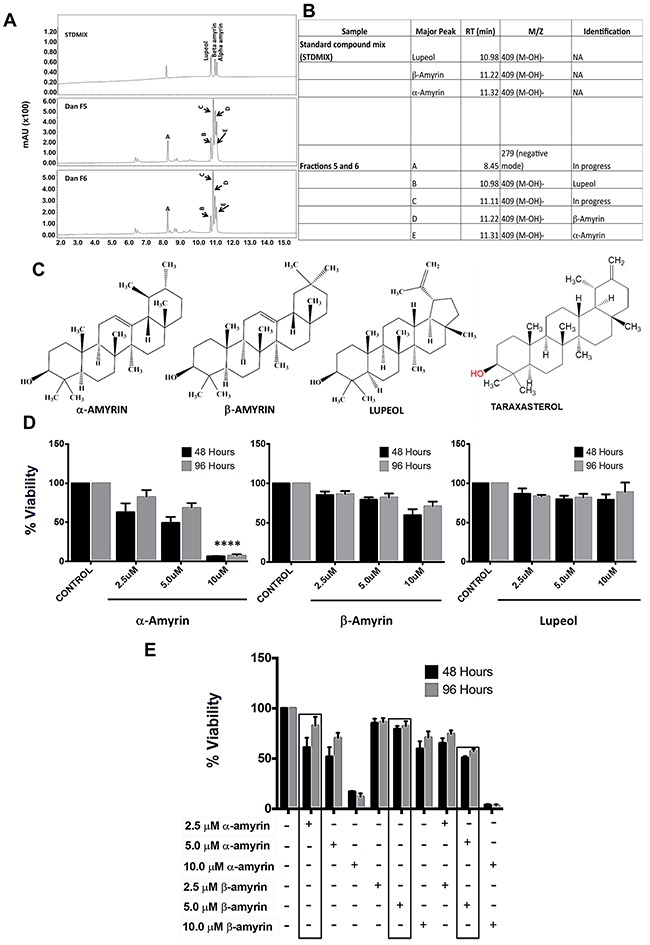
Identification of bioactive fractions & components within dandelion root extract **A.** UPLC-DAD-MS chromatogram of bioactive fraction #5 and #6, along with a standard mix of known compounds **B, C.** Identified compounds (by ChemDraw) in bioactive fractions #5 and #6. These triterpenes are isomers (426.7g/mol). **D.** Viability of HT-29 cells treated with α-amyrin, β–amyrin and lupeol ****P<0.0001 **E.** Compounds were analyzed alone and in combination with each other to assess combined effect in cancer cells. α-amyrin and β-amyrin showed an additive effect at slightly lower doses than the effective concentrations.

To commence the phytochemical analysis, dandelion root was extracted and fractionated using a silica gel column. The fractions obtained were then tested for bioactivity, by WST-1 cell viability assay. The results from the fraction studies were compared to those obtained from the bioactivity testing of the ethanolic extract of dandelion root. Bioactivity testing identified 6 out of 26 fractions with significant activity, comparable to the whole ethanolic extract. Following the identification of bioactive fractions, these active fractions were further fractionated to obtain secondary fractions (28) by a preparative HPLC column. Further bioactivity testing narrowed down the number of bioactive fractions. The identified bioactive fractions were analyzed by UPLC-DAD-MS, using a standard mix, to identify the different compounds within the fractions (Figure 9A). This led to the identification of four major components within two of the bioactive fractions (Fraction #5 and #6); α-amyrin, β-amyrin, lupeol and taraxasterol (Figure 9B – 9C). This data confirmed the presence of multiple bioactive components within DRE that could potentiate its cytotoxic activity against cancer cells.

#### Anti-cancer activity of identified bioactive compounds

Following the identification of 4 compounds within 2 of the bioactive fractions, the next phase of this study was to determine if any of these compounds had significant cytotoxicity to cancer cells, comparable to the DRE as a whole. Three of the four compounds are commercially available (α-amyrin, β-amyrin and lupeol). These compounds were tested alone in HT-29 cells, at increasing concentrations and for increasing time periods, and the viability of these cells was measured, using the WST-1 viability assay. We observed a decrease in the viability of cells treated with α-amyrin, with 10 μM as the most effective concentration. At the concentrations ≤ 10 μM, neither β-amyrin nor lupeol significantly reduced the viability of the cells (Figure 9D). To further test whether these components of the DRE could act in synergy, we tested their cytotoxicity in combination studies (α-amyrin + β-amyrin; α-amyrin + lupeol; β-amyrin + lupeol). Results showed that the viability reducing effect of α-amyrin and β-amyrin used in combination on HT-29 cells was additive, but not synergistic (Figure 9E). However, DRE is composed of multiple compounds, some of them might act synergistically and, collectively, are responsible for such strong and selective anti-cancer activity

#### Effect of DRE on the expression of cFLIP

**Figure 10:**
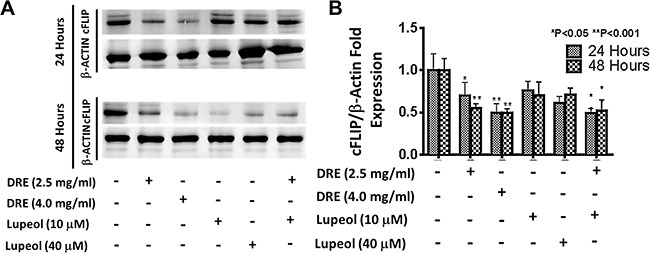
Dandelion root extract targets the extrinsic pathway of apoptosis by inhibiting the expression of cFLIP comparing the effect of DRE and lupeol on the expression of cFLIP Following treatment, cell lysates were obtained and proteins were separated on a gel. The proteins were transferred onto a PVDF membrane and probed for cFLIP levels, using β-actin as a loading control. Imaged blot of cFLIP expression levels and densitometry analysis of expression levels, compared to β-actin loading control.

Although lupeol did not significantly affect viability of HT-29 cells, there are studies indicating that lupeol plays a role in TRAIL-mediated apoptosis [42]. This study show that lupeol, at 40 μM, suppressed the expression of cellular FLICE-like inhibitory protein (cFLIP), an inhibitor of the extrinsic pathway of apoptosis, which prevents the formation of the death inducing signaling complex (DISC) and the subsequent activation of caspase-8 [43]. To further confirm the role of extrinsic pathway in DRE mediated apoptosis, we compared the effect of DRE to that of lupeol, in terms of their abilities to suppress the expression of cFLIP. Western blotting analysis showed that DRE, at 2.5 mg/ml, was sufficient to inhibit the expression of cFLIP in cancer cells (Figure 10). There was slightly additive effect when DRE was combined with the lower dose of lupeol, at 10 μM, again confirming the beneficial effect of multiple components within DRE, compared to the use of individual components. These results further confirmed the activity of DRE through the extrinsic pathway of apoptosis.

## DISCUSSION

This study shows, for the first time, the anti-cancer potential of aqueous dandelion root extract (DRE) in aggressive colon cancer models (*in-vitro* and *in-vivo*). Our results showed that DRE selectively reduced the metabolic activity of aggressive colon cancer cells, irrespective of their p53 status. DRE triggered apoptosis in colon cancer cells, without adverse effects on the viability and survival of non-cancerous colon mucosal epithelial cells. This effect was clearly distinct from the cells treated with the FOLFOX combinations. Furthermore, systemic administration of DRE was not toxic to mice drinking DRE-supplemented water for over a 3-month period. However, the 3 months oral administration of DRE significantly reduced the growth of colon tumors in xenograft models. These are novel findings revealing that DRE, in a dose and time dependent manner, selectively inhibits cancer cell growth. For further assessment of the efficacy of DRE as a preventative and therapeutic agent, transgenic mice models susceptible to the development of colorectal cancer or chemically induced colorectal cancer could be used. However, these results indicate that DRE and its anti-cancer components must be absorbed and circulated, in order to reach the site of the tumor (in order to inhibit tumor growth).

Some studies suggest that dandelion extracts have antioxidant, pro-oxidant and cytotoxic activity in colon adenocarcinoma cells, Caco-2, while being able to scavenge reactive oxygen species in mouse macrophage cells, RAW264.7 [44]. These effects on oxidative stress are attributed to the presence of flavonoids in the water and ethylacetate extracts. In this study, we confirmed the vulnerability of cancer cell mitochondria by showing that the DRE treatment led to a decrease in the mitochondrial membrane potential and increase in ROS levels in the isolated mitochondria. This was consistent with previous reports suggesting that the vulnerability of cancer cells' mitochondria stems from altered oxidative phosphorylation and reduced flux through the electron transport chain [45].

Secondly, we established that the DRE-induced destabilization of mitochondrial membranes was associated with a rapid activation of caspase-8. Interestingly, we observed that following DRE treatment, activated caspase-8 was released from the mitochondria into the cytoplasmic and peri-nuclear space, indicating that pro-caspase-8 may reside in the mitochondria and upon activation translocate to other locations in the cells, where they participate in the progression of apoptosis. These results correspond to those reported by Qin and colleagues [16].

Although caspase-8 was activated during the DRE exposure, it was not required for the progression of apoptosis in colon cancer cells, as the caspase-8 specific inhibitor, IETD-fmk, did not change the DRE response in these cells. This was in contrast to our previous study in leukemia and pancreatic cancer cells [12]. Clearly, the components of the DRE extract can stimulate/inhibit other signaling pathways in colon cancer cells and induce apoptosis, bypassing the requirement for caspase-8. Using a pathway finder array, we observed that DRE treatment induced the expression of several cell death genes, involved in both the apoptotic and autophagic cell death pathways in HT-29. These gene expression responses indicated that DRE could engage multiple signaling pathways to induce programed cell death (PCD) in colon cancer cells. Furthermore, we observed differential gene expression in HT-29 colorectal cancer cells and NCM460 normal colon mucosal epithelial cells, as genes that were up-regulated in the cancer cells, were down-regulated or not expressed in normal cells and vice versa.

Interestingly, in HT-29, the pro-survival genes, such as Bcl-2, GALNT5 and PARP-2, were down-regulated, while being up-regulated in NCM460. At the same time, the pro-apoptotic genes including Caspase-1, Interferon gamma and the TNF ligands and receptors, were up-regulated in HT-29 cells, prior to the apoptosis induction, while the same genes were down-regulated in NCM460 cells. Thus, the cancer cell-specific cytotoxicity of DRE must stem from its ability to involve multiple elements of cell death pathways, as evidenced by the complexity of the gene expression profiles.

We addressed the relevance of molecular complexity of the DRE extract to its anti-cancer properties. Phytochemical analysis and bioassays of the ethanolic dandelion root extract led to the identification of four pharmacologically active compounds, present in two out of the six bioactive fractions. These were α-amyrin, β-amyrin, lupeol and taraxasterol, 3 of which are commercially available - α-amyrin, β-amyrin, lupeol. At a high dose, ≥ 10 μM, α-amyrin reduced the viability of colon cancer cells; however, β-amyrin and lupeol did not. Furthermore, there were no synergistic interactions between these compounds and neither individually nor in combination would they display anti-cancer activity, comparable to that of the whole unfractionated DRE. Previous findings show that taraxasterol has anti-inflammatory and chemopreventive activity [46, 47], suggesting its importance in the anti-cancer activity of dandelion root extract, especially on the expression levels of COX-2. Additionally, we show that 10 μM lupeol is not very effective on its own. There is a slight decrease in the levels of cFLIP at this concentration but the effect was clearly noted at 40 μM, which is the published effective concentration for lupeol. At this concentration, lupeol can sensitize pancreatic cancer cells to TRAIL induced apoptosis, by inhibiting the expression of cFLIP an inhibitor of the extrinsic pathway of apoptosis [42]. As a result, it is understandable that we do not see a decrease in the viability of HT-29 cells with just 10 μM lupeol. In the present study, we have found that unfractionated DRE had a stronger effect on reducing the expression of cFLIP in HT-29, than lupeol alone. This was a dose and time dependent response. These results further confirmed that the anti-cancer activity of the DRE stemmed from its ability to activate the extrinsic pathway of apoptosis and even though the molecular steps of the pathway might be cell type specific, the multi-component composition of this extract is responsible for its overall bioactivity.

## CONCLUSIONS

Our results showed that aqueous dandelion root extract (DRE) efficiently and selectively triggers programmed cell death pathways in *in-vitro* and *in-vivo* colorectal cancer models. The results confirmed our hypothesis that the molecular complexity of the DRE extract is responsible for its anti-cancer activity, as it allows the engaging of multiple signaling pathways in cancer cells, including the mitochondria. Therefore, we can conclude that DRE, as a complex mixture might provide a complementary alternative to currently available chemotherapies. With these results, DRE is approved by Health Canada for Phase I clinical trials in hematological cancers. Their use might prove not only efficacious, but also associated with fewer and less severe side-effects and, thus, improve the quality of life and possibly increase the lifespan of cancer patients.

## MATERIALS AND METHODS

### Dandelion root extraction & preparation

The dandelion roots used for this study were obtained from Premier Herbal Inc. (Lot No. 318121). The root extract was prepared using a previously published protocol with the following modifications [11]. Dried dandelion root was immersed in liquid nitrogen for about 5 to 10 minutes, until thoroughly frozen. The frozen pieces were ground up in an impingement grinder to an average particle size of ≤ 45 μm. Following grinding, dandelion root powder was extracted in boiling water on low heat for 3 hours. The total extracted material was filtered through a NITEX nylon mesh filter (LAB PAK; Sefar BDH Inc. Chicoutini, Quebec CA) and the filtrate was spun down at 800 × *g* for 5 minutes at room temperature. The supernatant was filtered through a 0.45 μm filter, followed by lyophilization. The dried extracted material was reconstituted in water to give a final stock solution of 100 mg/ml and then passed through a 0.22 μm filter, in a biological safety cabinet and stored at 4°C or −20°C for long term storage. This material was used for all the experiments described in this study.

### Cell culture and treatment

Human colon cancer cell lines HT-29 (p53 mutant) and HCT116 (p53 WT) (Muller et al., 2013) were purchased from ATCC (Manassas, VA). These cells were cultured in McCoy's 5a medium (ATCC, Catalog no. 30-2007), supplemented with 10% fetal bovine serum (FBS) and 40 μg/ml gentamicin (Life Technologies, Mississauga, ON). Normal human colon mucosal epithelial cell line (NCM460, Incell Corporation, LLC, San Antonio TX) was sub-cultured in RPMI 1640 medium (Sigma Aldrich, Mississauga, ON), supplemented with 10% FBS and 40 μg/ml gentamicin. These cells were grown and maintained in an incubator, set at 37°C, with an atmosphere containing 5% CO_2_ and 95% humidity.

To assess the efficacy of DRE in our cell culture models, cells were plated and grown to 50 – 70% confluence prior to treatment with dandelion root extract (DRE), at increasing concentration (0.5 mg/ml – 6.0 mg/ml). Subsequent to treatment, cells were analyzed for efficacy of DRE, as described below. All cells were cultured for ≤ 4 months, before being discarded and fresh frozen cells were used to continue studies, lasting longer than the 4-month period.

### Assessment of cellular metabolic activity & viability

To examine the viability of colon cancer and normal colon mucosal epithelial cells after treatment, cells were incubated with cell proliferation reagent WST-1 (Catalog No. 05 015 944 001, Roche Diagnostics) for 4 hours at 37°C, following treatment with DRE at indicated doses and time points, using the manufacturers protocol. Absorbance readings of the formazan product were obtained 450 nm using a spectrofluorometer (SpectraMax Gemini XS, Molecular Devices, Sunnyvale, CA) Viability readings were analyzed using GraphPad Prism 6.0 288 software and expressed as a percentage of the control untreated groups.

### Assessment of programmed cell death induction

This distinctive externalization of phosphatidylserine on the external membrane of apoptotic cells was exploited to assess the induction of apoptosis, following DRE treatment, according to a previously published protocol [13, 48]. Following treatment with DRE, cells were trypsinized (0.15% trypsin) to lift adherent cells from the plates. The cells were washed twice in phosphate buffered saline (PBS) and resuspended in Annexin-V binding buffer (10 mM HEPES, 10 mM NaOH, PH 7,5, 140 mM NaCl, 2.5 mM CaCl_2_ and 50nM sucrose) and Annexin-V Alexa Fluor 488 conjugate (Catalog No. A13201, Life Technologies, Burlington, ON), which binds to the exposed phosphatidylserine, at a 1:50 ratio, with respect to the binding buffer. This reaction was incubated at room temperature for 15 minutes. Hoechst 33342, a photosensitive DNA binding dye (Catalog No. H1399; Life technologies), was used as a counterstain, at a final concentration of 10 μM, in the last 10 minutes of the incubation at room temperature. At the time of Hoechst staining, cells were counterstained with propidium iodide, a cell impermeable photosensitive DNA binding dye (Catalog No. P4170, Sigma Aldrich, Mississauga, ON), at a final concentration of 1 μg/ml. Following the incubation, cells were visualized and images were obtained using a fluorescence microscope (Leica DMI 6000 fluorescence microscope, with a Leica DFC 360FX camera and Leica STP6000 control board).

The images were obtained at 400X magnification and fluorescence quantification was also carried out using a TALI image-based cytometer (Catalog No. T10796, Life Technologies, Burlington ON), using a previously published protocol [49, 50].

### Wound-healing migration assay

HT-29, HCT116 and NCM460 cells were grown to 80% of confluence in 6-well tissue culture plates (Catalog No. 83.3920.300, Sarstedt) and “wounded” with a sterile 200 μL pipet tip to remove cells. PBS washing was used to remove loosely attached cells before treatment. Cells were pre-treated with thymidine for 18 hours to halt proliferation, following which cells were treated with DRE. The progression of migration was photographed at different times (0 – 48 hours) following staining with Hoechst 33342, under a light and fluorescent microscope. The area of invasion and the distance of wound healing closure by the migrated cells was measured.

### Evaluation of mitochondrial membrane potential

JC-1 (5,5′, 6,6′-tetrachloro-1,1′, 3,3′-tetraethylbenzamidazolylcarbocyanine iodide) mitochondrial membrane potential kit (Catalog No. M34152, Life Technologies, Burlington ON) was used to monitor membrane destabilization in cells undergoing apoptosis. In apoptotic and necrotic cells, with defective mitochondria, JC-1 only exists in its monomeric form, diffused in the cytosol, with no accumulation in the mitochondria, leading to a distinct lack of red fluorescence. Treated cells were incubated with JC-1 dye, at a final concentration of 200 nM, for 45 minutes at 37°C. The cells were counterstained with Hoechst 33342 and were examined by fluorescent microscopy and fluorescent images were taken at 400X magnification. Samples were also quantified using image-based cytometry.

### Mitochondrial isolation and measurement of ROS production

Mitochondria were isolated from untreated HT-29 and NCM460 cells to investigate reactive oxygen species (ROS) production. Cells were washed twice in cold PBS, resuspended in hypotonic buffer (1 mM EDTA, 5 mM Tris-HCl, 210 mM mannitol, 70 mM sucrose, 10 μM Leu-pep, 10 μM Pep-A and 100 μM PMSF) and immediately homogenized, before centrifuging at 600 × *g* for 5 minutes, at 4°C to get rid of nuclear pellet and unbroken cell debris. The resulting cytosolic supernatant was centrifuged again at 10,000 × *g* to obtain a mitochondrial pellet, which was resuspended in cold hypotonic buffer.

To measure the production of ROS, Amplex Red (Catalog No. A12222, Life Technologies) was used. Following resuspension in cold hypotonic buffer, ≥ 20 μg of protein was added to wells of a 96-well opaque plate. Treatment was done with 2.5 mg/ml DRE, a positive control, 250 μM paraquat (PQ) (Catalog No. 856177, Sigma Aldrich, Mississauga ON) and a negative control, 3 mM N-Acetylcysteine (N-AC) (Catalog No. A7250, Sigma Aldrich, Mississauga ON). Amplex Red reagent was added to each well at a final concentration of 50 μM; horseradish peroxidase (HRP) was added in the ratio of 6 units per 200 μL. Fluorescence readings were taken every 5 minutes for a total time of 5 hours at Ex. 560 and Em. 590 nm on a spectrofluorometer (SpectraMax Gemini XS, Molecular Devices, Sunnyvale, CA). The readings were analyzed on GraphPad Prism 6.0 288 software and expressed as relative fluorescence units (RFU) per μg protein.

### Evaluation of caspase activation

Cysteine-Aspartic Proteases (Caspases) are major players in the initiation and execution of apoptosis [13]. Caspase activation was measured using an Image-iT Live caspase detection kit (Catalog No. I35105, Life Technologies, Mississauga ON). Following treatment for an hour with DRE, cells were stained with a cell permeable FLICA dye for the detection of active caspases, at 150X dilution and incubated for 45 minutes at 37°C. Following incubation with the dye, cells were washed in 1X wash buffer and counterstained with propidium iodide. The stained cells were visualized by TALI image-based cytometry. To further confirm the role of caspases in apoptosis induction by DRE, a caspase-8 specific inhibitor, IETD-fmk (Catalog No. 218759, EMD4Biosciences, San Diego, CA) or a pan-caspase inhibitor, ZVAD-fmk (Catalog No. 219007, EMD4Biosciences) were incubated with HT-29 cells, an hour before DRE treatment, as described above. Cells were analyzed for metabolic viability by the WST-1 viability assay.

### Immunocytochemical analysis of caspase activation

To confirm the activation of caspases and their localization upon activation, HT-29 cells were plated onto poly-L-lysine (Catalog No. P4707, Sigma Aldrich, Canada) coated coverslips in a 6-well plate and allowed to attach to the coverslips for 24 hours. These cells were then treated with DRE for different time points, ranging from 30 minutes to 24 hours, following which the cells were incubated with a mitochondrial specific due, MitoTracker (Invitrogen, Canada) for 45 minutes, before the cells were obtained for immunocytochemical analysis. Cells were fixed in 3.7% paraformaldehyde (PFA) solution for 5 minutes at room temperature, followed by a 1.4% formaldehyde/0.1% NP-40 solution for 1.5 minutes at room temperature. Subsequent to fixation, cells were incubated with a blocking solution of 5% goat serum in PBS, followed by incubation overnight at 4°C with anti-mouse primary antibody, specific to active caspase-8 (Santa Cruz Biotechnology, CA). The following day, coverslips were incubated with a goat anti-mouse secondary antibody, conjugated to AlexaFluor 488 (CellSignalling Technology, MA) for an hour at room temperature. The coverslips were washed in PBS and incubated with Hoechst 33342 for 10 minutes, at a final concentration of 10 μM and mounted on slides, using 80% glycerol. The slides were stored at 4°C until ready for visualization by fluorescent microscopy.

### Gene expression profiling of DRE treated cells:

#### RNA extraction and cDNA synthesis

Following treatment of HT-29 and NCM460 cells, with DRE, total cellular RNA was extracted using the Qiagen RNeasy Mini Kit (Qiagen, Inc.), according to the manufacturer's protocol. The RNA quality was examined using gel electrophoresis and measuring the A280/A260 ratio (NanoDrop 2000). The cDNA was synthesized from 500 ng of total RNA by using the RE3 Reverse Transcriptase Mix first-strand synthesis system. Following a denaturation step of 5 minutes at 42°C, RNA was reverse transcribed to a single stranded cDNA using oligo(dT) primers (Qiagen, Inc.). The reverse transcription reaction was performed in a total volume of 20 μL at 42°C for 15 minutes, immediately followed by 95°C for 90 minutes.

#### PCR array (cell death signaling pathway)

The polymerase chain reaction (PCR) for the cell death signaling pathway was performed, following the reverse transcription of isolated RNA, using the RT^2^Profiler PCR array system from Qiagen, Inc. The PCR array was performed to combine the quantitative performance of the SYBR Green based system, with multiple profiling abilities of the pathway-focused gene expression [51]. Gene specific primer sets for 84 relevant genes in the major programmed cell death pathways (Apoptosis, Autophagy and Programmed Necrosis) wee contained in 384-well (4 × 96 wells) plates. Experimental controls included 5 housekeeping genes, a positive control and a negative control gene (Catalog No. PAHS-212Z, Qiagen, Inc. Toronto, ON).

Amplification of specific gene products was detected using the SYBR Green PCR mastermix and the real time amplification data was gathered using the ABI 7900HT software. The samples were amplified for 40 cycles for 15 s at 95°C and 60 s at 60°C. Each curve was completed with a melting curve analysis, to confirm the specificity of amplification. Gene expression was normalized to internal controls (housekeeping genes) to establish fold change in gene expression between the controls and treated samples by ΔΔC_T_ method (Qiagen RT^2^ Profiler PCR Array Analysis program).

### Western blotting analysis

SDS-PAGE was performed on the protein samples. Treated cells were lysed in lysis buffer (0.1% NP40, 20 mM Tris-HCl, 100 mM NaCl and 5 mM EDTA), following which, the total protein was measured by Bradford assay. Proteins were separated on a 10% or 12% gel (depending on the molecular weights of the proteins of interest) and then transferred to a nitrocellulose membrane. Following transfer, the membranes were blocked in a milk solution (5% w/v milk in Tris-Buffered Saline with Tween-20 (TBST)) or BSA solution (5% w/v BSA in TBST) for 90 minutes. Subsequent to the blocking step, membranes were probed with primary antibodies, overnight at 4°C; anti-TMEM57, raised in rabbit (1:2000), (Catalog No. NBP1-84605), anti-ULK1, raised in rabbit (1:1000) (Catalog No. NBP2-24738), anti-p62, raised in mouse (1:1000), (Catalog No. H00008878M01), anti-Bcl-2A1, raised in rabbit (1:1000), (Catalog No. NBP1-76715), anti-Bcl-xL, raised in rabbit (1:2000), (Catalog No. NB100-81814), anti-PARP-2, raised in rabbit (1:1000) (Catalog No. NB100-185), anti-ERK1/2, raised in rabbit (1:1000), (Catalog No. NB100-82099) and anti-Grb 2, raised in rabbit (1:1000), (Catalog No. NB110-57013). The aforementioned antibodies were obtained from Novus Biologicals, Oakville ON. Anti-BID, raised in rabbit (1:1000), (Catalog No. GTX110568), anti-COX-2, raised in mouse (1:2000), (Catalog No. GTX61755) and anti-cFLIP, raised in rabbit (1:1000), (Catalog No. GTX28421) were obtained from GeneTex Inc. Irvin, CA and anti-actin, raised in mouse (1:1000), (Catalog No. sc-81178) was obtained from Santa Cruz Biotechnology, CA. Following incubation with the primary antibodies, membranes were washed in TBST (1X – 15 minute wash and 2X – 5 minute wash) and following the washes, membranes were incubated with anti-mouse or anti-rabbit horseradish peroxidase conjugated secondary antibody (1:1000) – (Catalog No. ab6728 Abcam, Cambridge MA and HAF008, Novus Biologicals, Oakville ON, respectively) for an hour at room temperature. The membranes were then washed for 3 × 5 minutes in TBST, followed by band visualization with a Visiglo Select HRP chemiluminescent substrate kit (Catalog No. CA11027-138, VWR International, Mississauga ON). Densitometry analyses were performed using Image J software.

### Fractionation & phytochemical analysis of dandelion root extract

Bioactivity analyses of primary fractions (supplementary materials & methods) led to the identification of 5 bioactive fractions, which were further fractionated to yield 24 secondary sub-fractions. Pre-fractionation of secondary fractions with significant bioactivity was carried out and separated on a preparative scale 1200 series Agilent preparative scale HPLC using a reversed-phase Gemini Axia 250 × 21.2 mm column, particle size 10 μm (Phenomenex Inc., Torrance CA), using an isocratic mobile phase composition of 45% THF in 55% water at 37.5mL.min to afford uvaol (12.0 mg, 0.0008%), betulin, (100 mg, 0.007%), α-amyrin (6.5 mg, 0.0004%), and betulinic acid (5.0 mg, 0.0003%) at the monitoring wavelength of 210 nm, bandwith 4, reference off.

The final preparative scale isolation of the phytochemicals was undertaken using a reversed-phase Gemini Axia 250 × 21.2 mm column, particle size 10 μm (Phenomenex Inc., Torrance CA), on an Agilent 1200 Series preparative HPLC system comprising a binary pump, an autosampler with a 2 mL loop, a diode array detector, with a flow cell (Path length 3 mm and maximum pressure limit, 120 bar), and a fraction collector (40 μL collection tubes). IR spectra were recorded on a Shimadzu 8400-S FT/IR spectrometer. Optical rotations were registered on a Perkin Elmer 241 digital polarimeter. NMR spectra were recorded on a Bruker Avance 400 spectrometer in C5D5N, at either 400 MHz (1H) or 100 (13C) MHz, using tetramethylsilane (TMS) as an internal standard. EIMS and HREIMS were obtained on a Kratos Concept IIH mass spectrometer.

### In-vivo assessment of dandelion root extract

#### Toxicity assessment

Six week old Balb/C mice were obtained from Charles River Laboratories and housed in constant laboratory conditions of 12-hour light/dark cycle, in accordance with the animal protocols outlined in the University of Windsor research ethics board (AUPP #10-17). Following acclimatization, mice were divided into two groups (5 animals/untreated control group and 4 animals/treatment group). The control untreated group was orally gavaged with PBS, with a volume corresponding to the body weight of each animal, while the second group was orally gavaged with 40 mg/kg/day of dandelion root extract in water respectively for 75 days; animals were gavaged 3 times a week. For the duration of the study, toxicity was measure by weighing mice twice a week and urine was collected for protein urinalysis by urine dipstick and Bradford assays. Following the duration of the study, mice were sacrificed and their organs (livers, kidneys and hearts) were obtained for immunohistochemical and toxicological analyses, by Dr. A. Brooke at the University of Guelph.

#### Efficacy of DRE in tumor xenograft models of immunocompromised mice

Six week old make CD-1 nu/nu mice were obtained from Charles River Laboratories and housed in constant laboratory conditions of 12-hour light/dark cycle, in accordance with the animal protocols. Following acclimatization, mice were subcutaneously injected in the right and left hind flanks with a colon cancer cell suspension (in PBS) at a concentration of 2 × 10^6^ cells/ mouse (HT-29, p53^−/−^ in the left flank and HCT116, p53^+/+^ in the right flank). Tumors were allowed to develop (approximately a week), following which, the animals were randomized into treatment groups of 4 mice per group, an untreated control group and a DRE treatment group, as outlined in the toxicity studies above. The mice were orally gavaged with either PBS or DRE in PBS at 40 mg/kg/day for 75 days. All mice were assessed for toxicity, as well as efficacy of oral administration on the growth of tumors. The tumors were assessed every other day by measuring the length, width and height, using a standard caliper and the tumor volume was calculated according to the formula π/6 × length × width. The mice were also assessed for any weight loss for the duration of the study, which also lasted 75 day. Following the study, mice were sacrificed and their organs and tissues (livers, kidneys, hearts and tumors) were obtained and stored in formaldehyde for immunohistochemical and toxicological analysis.

### Hematoxylin & eosin (H & E) staining

Mice organs were fixed in 10% formaldehyde in PBS, following which the organs were cryosectioned into 10-micron sections and placed on a superfrost/Plus microscope slides (Fisherbrand, Fisher Scientific). Sections of organs were stained according to a standardized H & E protocol [52].

### Statistical analyses

All experiments were repeated at least three independent times. Statistical analysis was performed using GraphPad Prism 6.0 288 software. Statistical tests included the Students T-test and Two-way Anova.

## References

[1] Jemal A, Bray F, Ferlay J (2011). Global Cancer Statistics. CA Cancer J Clin.

[2] Siegel R, Naishadham D, Jemal A (2013). Cancer Statistics, 2013. CA Cancer J Clin.

[3] Mann J (2002). Natural products in cancer chemotherapy: past, present and future. Nature Reviews, Cancer.

[4] Ganesan A (2008). The impact of natural products upon modern drug discovery. Current Opinion in Chemical Biology.

[5] Newman DJ, Cragg GM (2012). Natural products as sources of new drugs over the 30 years from 1981 to 2010. Journal of natural products.

[6] Foster BC, Arnason JT, Briggs CJ (2005). Natural health products and drug disposition. Annual Review of Pharmacology and Toxicology.

[7] Nobili S, Lippi D, Witort E, Donnini M, Bausi L, Mini E, Capaccioli S (2009). Natural compounds for cancer treatment and prevention. Pharmacological Research.

[8] Yarnell E, Abascal K (2009). Dandelion (Taraxacum officinale and T mongolicum). Integrative Medicine.

[9] Chatterjee SJ, Ovadje P, Mousa M, Hamm C, Pandey S (2011). The Efficacy of Dandelion Root Extract in Inducing Apoptosis in Drug-Resistant Human Melanoma Cells. Evidence-Based Complementary and Alternative Medicine.

[10] Ovadje P, Chatterjee S, Griffin C, Tran C, Hamm C, Pandey S (2011). Selective induction of apoptosis through activation of caspase-8 in human leukemia cells (Jurkat) by dandelion root extract. Journal of Ethnopharmacology.

[11] Ovadje P, Chochkeh M, Akbari-Asl P, Hamm C, Pandey S (2012a). Selective Induction of Apoptosis and Autophagy Through Treatment With Dandelion Root Extract in Human Pancreatic Cancer Cells. Pancreas.

[12] Ovadje P, Hamm C, Pandey S (2012b). Efficient induction of extrinsic cell death by dandelion root extract in human chronic myelomonocytic leukemia (CMML) cells. PloS One.

[13] Fadeel B, Orrenius S (2005). Apoptosis: a basic biological phenomenon with wide-ranging implications in human disease. Journal of Internal Medicine.

[14] Loo G, Saelens X, Matthijssens F, Schotte P, Beyaert R, Declercq W, Vandenabeele P (2002). Caspases are not localized in mitochondria during life or Death. Cell Death and Diseases.

[15] Chandra D, Choy G, Deng X, Bhatia B, Daniel P, Tang D (2004). Association of Active Caspase 8 with the Mitochondrial Membrane during Apoptosis: Potential Roles in Cleaving BAP31 and Caspase 3 and Mediating Mitochondrion-Endoplasmic Reticulum cross talk in etoposide-induced cell death. Molecular Cell Biology.

[16] Qin Z, Wang Y, Kikly K, Sapp E, Kegel K, Aronin N, DiFiglia M (2001). Pro-caspase-8 Is Predominantly Localized in Mitochondria and Released into Cytoplasm upon Apoptotic Stimulation. Journal of Biological Chemistry.

[17] Lu Z, Xu S (2006). ERK1/2 MAP Kinases in Cell Survival and Apoptosis. IUBMB LIFE.

[18] Mebratu Y, Tesfaigzi Y (2009). How ERK1/2 Activation Controls Cell Proliferation and Cell Death Is Subcellular Localization the Answer?. Cell Cycle.

[19] Peek RM (2004). Prevention of colorectal cancer through the use of COX-2 selective inhibitors. Cancer Chemotherapy and Pharmacology.

[20] Tsujimoto Y, Croce CM (1986). Analysis of the structure, transcripts, and protein products of bcl-2, the gene involved in human follicular lymphoma. Proceedings of the National Academy of Sciences.

[21] Vogler M (2012). BCL2A1: the underdog in the BCL2 family. Cell Death Differ.

[22] Banchereau J, Bazan F, Blanchard D, Briè F, Galizzi JP, van Kooten C, Liu YJ, Rousset F, Sealand S (1994). The CD40 Antigen and its Ligand. Annual Review of Immunology.

[23] He H, Shen Z, Zhang H, Wang X, Tang Z, Xu J, Sun Y (2014). Clinical significance of polypeptide N-acetylgalactosaminyl transferase-5 (GalNAc-T5) expression in patients with gastric cancer. Br J Cancer.

[24] Liang YC, Hsu CY, Yao YL, Yang WM (2013). PARP-2 regulates cell cycle-related genes through histone deacetylation and methylation independently of poly(ADP-ribosyl)ation. Biochemical and Biophysical Research Communications.

[25] Yélamos J, Schreiber V, Dantzer F (2014). Toward specific functions of poly(ADP-ribose) polymerase-2. Trends in Molecular Medicine.

[26] Schalk JAC, Offenberg HH, Peters E, Groot NPB, Hoovers JMN, Heyting C (1999). Isolation and characterization of the human SCP2 cDNA and chromosomal localization of the gene. Mammalian Genome.

[27] Pike L, Singleton DC, Buffa F, Abramczyk O, Phadwal K, Li J, Simon AK, Murray JT, Harris AL (2013). Transcriptional up-regulation of ULK1 by ATF4 contributes to cancer cell survival. Biochem J.

[28] Russell RC, Tian Y, Yuan H, Park HW, Chang YY, Kim J, Guan KL (2013). ULK1 induces autophagy by phosphorylating Beclin-1 and activating VPS34 lipid kinase. Nat Cell Biol.

[29] Grespi F, Soratroi C, Krumschnabel G, Sohm B, Ploner C, Geley S, Villunger A (2010). BH3-only protein Bmf mediates apoptosis upon inhibition of CAP-dependent protein synthesis. Cell Death Differ.

[30] Hausmann M, Leucht K, Ploner C, Kiessling S, Villunger A, Becker H, Rogler G (2011). BCL-2 Modifying Factor (BMF) Is a Central Regulator of Anoikis in Human Intestinal Epithelial Cells. Journal of Biological Chemistry.

[31] Donald CD, Sun CQ, Lim SD, Macoska J, Cohen C, Amin MB, Petros JA (2003). Cancer-Specific Loss of [bgr]-Defensin 1 in Renal and Prostatic Carcinomas. Lab Invest.

[32] Hu C, Kitts DD (2003). Antioxidant, Prooxidant, and Cytotoxic Activities of Solvent-Fractionated Dandelion (Taraxacum officinale) Flower Extracts in vitro. Journal of Agriculture Food Chemistry.

[33] Smirnov DA, Brady L, Halasa K, Morley M, Solomon S, Cheung VG (2012). Genetic variation in radiation-induced cell death. Genome Research.

[34] Schroder K, Hertzog PJ, Ravasi T, Hume DA (2004). Interferon-γ: an overview of signals, mechanisms and functions. Journal of Leukocyte Biology.

[35] Kaltschmidt B, Kaltschmidt C, Hofmann TG, Hehner SP, Dröge W, Schmitz ML (2000). The pro- or anti-apoptotic function of NF-κB is determined by the nature of the apoptotic stimulus. European Journal of Biochemistry.

[36] Stark LA, Reid K, Sansom OJ, Din FV, Guichard S, Mayer I, Dunlop MG (2007). Aspirin activates the NF-kappaB signalling pathway and induces apoptosis in intestinal neoplasia in two in vivo models of human colorectal cancer. Carcinogenesis.

[37] Schall TJ, Lewis M, Koller KJ, Lee A, Rice GC, Wong GHW, Goeddel DV (2014). Molecular cloning and expression of a receptor for human tumor necrosis factor. Cell.

[38] Denes A, Lopez-Castejon G, Brough D (2012). Caspase-1: is IL-1 just the tip of the ICEberg?. Cell Death & Disease.

[39] Devine MJ, Plun-Favreau H, Wood NW (2011). Parkinson's disease and cancer: two wars, one front. Nat Rev Cancer.

[40] Bethge N, Lothe RA, Honne H, Andresen K, Trøen G, Eknæs M, Lind GE (2014). Colorectal cancer DNA methylation marker panel validated with high performance in Non-Hodgkin lymphoma. Epigenetics.

[41] Hassan M, Watari H, AbuAlmaaty A, Ohba Y, Sakuragi N (2014). Apoptosis and Molecular Targeting Therapy in Cancer. BioMed Research International.

[42] Nascimbeni AC, Fanin M, Masiero E, Angelini C, Sandri M (2012). The role of autophagy in the pathogenesis of glycogen storage disease type II (GSDII). Cell Death Differ.

[43] Murtaza I, Saleem M, Adhami VM, Hafeez BB, Mukhtar H (2009). Suppression of cFLIP by lupeol, a dietary triterpene, is sufficient to overcome resistance to TRAIL-mediated apoptosis in chemoresistant human pancreatic cancer cells. Cancer Research.

[44] Safa AR (2012). c-FLIP, a master anti-apoptotic regulator. Experimental Oncology.

[45] Hu C, Kitts DD (2005). Dandelion (Taraxacum officinale) flower extract suppresses both reactive oxygen species and nitric oxide and prevents lipid oxidation in vitro. Phytomedicine.

[46] Ralph SJ, Neuzil J (2009). Mitochondria as targets for cancer therapy. Molecular Nutrition & Food Research.

[47] Ovesna Z, Vachalkova A, Horvathova K (2004). Taraxasterol and b-sitosterol: New naturally compounds with chemoprotective/chemopreventive effects. Neoplasma.

[48] Zhang X, Xiong H, Liu L (2012). Effects of taraxasterol on inflammatory responses in lipopolysaccharide-induced RAW 264.7 macrophages. Journal of Ethnopharmacology.

[49] Rieger AM, Nelson KL, Konowalchuk JD, Barreda DR (2011). Modified annexin V/propidium iodide apoptosis assay for accurate assessment of cell death. Journal of Visualized Experiments.

[50] Remple K, Stone L (2011). Assessment of GFP expression and viability using the tali image-based cytometer. Journal of Visualized Experiments: JoVE.

[51] Chan L, Wilkinson A, Paradis B, Lai N (2012). Rapid Image-based Cytometry for Comparison of Fluorescent Viability Staining Methods. Journal of Fluorescence.

[52] Fischer AH, Jacobson KA, Rose J, Zeller R (2008). Hematoxylin and eosin staining of tissue and cell sections. Cold Spring Harbor Protocols.

